# The Landscape of *Pseudomonas aeruginosa* Membrane-Associated Proteins

**DOI:** 10.3390/cells9112421

**Published:** 2020-11-05

**Authors:** Sara Motta, Davide Vecchietti, Alessandra M. Martorana, Pietro Brunetti, Giovanni Bertoni, Alessandra Polissi, Pierluigi Mauri, Dario Di Silvestre

**Affiliations:** 1 National Research Council-Institute for Biomedical Technologies, F.lli Cervi 93, 20090 Segrate (Milan), Italy; sara.motta1984@gmail.com (S.M.); gunzapper@gmail.com (P.B.); pierluigi.mauri@itb.cnr.it (P.M.); 2 Department of Biosciences, University of Milan, Celoria 26, 20133 Milan, Italy; vecchietti.dav@gmail.com (D.V.); giovanni.bertoni@unimi.it (G.B.); 3 Department of Pharmacological and Biomolecular Sciences, University of Milan, Balzaretti, 20133 Milan, Italy; alessandra.martorana@unimi.it (A.M.M.); alessandra.polissi@unimi.it (A.P.)

**Keywords:** *Pseudomonas aeruginosa*, MudPIT, membrane proteins, shaving, exposed peptides

## Abstract

Background: *Pseudomonas aeruginosa* cell envelope-associated proteins play a relevant role in infection mechanisms. They can contribute to the antibiotic resistance of the bacterial cells and be involved in the interaction with host cells. Thus, studies contributing to elucidating these key molecular elements are of great importance to find alternative therapeutics. Methods: Proteins and peptides were extracted by different methods and analyzed by Multidimensional Protein Identification Technology (MudPIT) approach. Proteomic data were processed by Discoverer2.1 software and multivariate statistics, i.e., Linear Discriminant Analysis (LDA), while the Immune Epitope Database (IEDB) resources were used to predict antigenicity and immunogenicity of experimental identified peptides and proteins. Results: The combination of 29 MudPIT runs allowed the identification of 10,611 peptides and 2539 distinct proteins. Following application of extraction methods enriching specific protein domains, about 15% of total identified peptides were classified in trans inner-membrane, inner-membrane exposed, trans outer-membrane and outer-membrane exposed. In this scenario, nine outer membrane proteins (OprE, OprI, OprF, OprD, PagL, OprG, PA1053, PAL and PA0833) were predicted to be highly antigenic. Thus, they were further processed and epitopes target of T cells (MHC Class I and Class II) and B cells were predicted. Conclusion: The present study represents one of the widest characterizations of the *P. aeruginosa* membrane-associated proteome. The feasibility of our method may facilitates the investigation of other bacterial species whose envelope exposed protein domains are still unknown. Besides, the stepwise prioritization of proteome, by combining experimental proteomic data and reverse vaccinology, may be useful for reducing the number of proteins to be tested in vaccine development.

## 1. Introduction

*Pseudomonas aeruginosa* is a highly adaptable Gram-negative bacterium able to infect several hosts including human beings [1]. Since it is an opportunistic pathogen, the occurrence of antimicrobial resistance makes its treatment difficult [2]. This is due to different reasons, ranging from the production of a wide variety of virulence factors [3] to the role of membrane proteins, enzymes and other molecules that protect bacterial cells from antibiotics and host immune system [4]. In this scenario, a relevant role is played by the complex architecture of the *Pseudomonas aeruginosa* envelope. It includes an inner cytoplasmic membrane (IM) and an additional asymmetric lipid bilayer, the outer membrane (OM), that act as selective barrier [5]. Moreover, surface-exposed proteins represent the first molecules involved in the interaction with pathogens, playing a relevant role to invade host cells. Thus, OM-exposed proteome has the potential to be an attractive drug target for Gram-negative bacteria, and in particular for those highly infective like *P. aeruginosa* [6].

Due to the relevant role of the envelope-associated proteins, in the last two decades different studies aimed to characterize the *P. aeruginosa* envelope proteome. Most of them focused on OM [7,8,9,10] and global membrane [11,12,13], while a lesser number analyzed proteins from periplasmic space [14] and IM [15]. Some of these studies were based on two-dimensional (2-D) gel electrophoresis protein separation [7,8,10,14]. On the contrary, other authors used liquid chromatography (LC) coupled to mass spectrometry (MS) with the purpose for a large scale protein identification [9,11,12,15].

To improve knowledge about *P. aeruginosa* cell envelope, we here analyzed the *P. aeruginosa* membrane-associated proteome by combining selective membrane, peptide and protein extraction methods [9,16] with a high-throughput proteomic approach based on LC-MS [17]. Besides, to provide a landscape of *P. aeruginosa* membrane-anchored proteins, we discriminated inner from outer membrane proteins, as well as trans-membrane from surface-exposed peptides by applying multivariate statistical analysis [18]. Finally, since surface-exposed classified proteins may represent a valuable source of information useful in developing new vaccines and drugs against *P. aeruginosa*, a preliminary classification of the putative antigenic and immunogenic epitopes (MHC class I, MHC class II and B cell) was also provided.

## 2. Material and Methods

### 2.1. Bacterial Growth Conditions and Membrane Fractionation

*Pseudomonas aeruginosa* PAO1 was grown in Mueller Hinton (MH, SigmaAldrich) broth at 37 °C until cells reached OD600 0.8. A total amount of 200 OD600 of cells were processed as described in Lo Sciuto et al. [19]. The IM and OM of *Pseudomonas aeruginosa* PAO1 were separated by sucrose gradient ultracentrifugation according to published procedures with some modification [19]. Briefly, total membranes were resuspended in 1 mL of 10 mM Tris/HCl buffer (pH 7.8) containing 25% (*w*/*v*) sucrose and 800 μL were layered on top of the sucrose gradient. Fractions (400 μL) were collected from the top and 50 μL each was assayed for NADH oxidase activity. The distribution profile of LPS was estimated by western blot using the anti-*Pseudomonas aeruginosa* outer core specific (MEDIBAS).

### 2.2. Membrane Proteins Trypsin Digestion

The pellet contaning the global membrane fraction (GMF) was washed in sequence with PBS, 1 M NaCl and distilled water to remove contaminants. To enrich the surface exposed peptides, the isolated membranes were shaved; in detail, the membranes were resuspended in PBS buffer and subsequently treated overnight with 40 μg/mL trypsin (Sequencing Grade Modified Trypsin, Promega, Madison, WI, USA) at 37 °C under stirring. The digested peptides were isolated through ultracentrifugation (135,000× *g* for 60 min at 4 °C), collected and finally analyzed by Multidimensional Protein Identification Technology (MudPIT), (*n* = 6).

To extract the proteins inserted into the membranes, IM (*n* = 6) and OM (*n* = 5) fractions were resuspended and denaturated by a saline buffer containing 2% sarkosyl detergent (SigmaAldrich). The total protein content was quantified using the SPNTM-Protein Assay (G-Biosciences, St Louis, MO, USA) and 50 μg per sample were treated with RapiGestTM SF (Waters Corporation, Milford, MA, USA) at the final concentration of 0.2% (*w*/*v*). After incubation at 100 °C for 5 min, the samples were cooled to room temperature. Trypsin (Sequencing Grade Modified Trypsin, Promega, Madison, WI, USA) was added to each sample at an enzyme/substrate ratio of 1:50 (*w*/*w*) and incubated at 37 °C overnight; then, another aliquot of enzyme was added at an enzyme/substrate ratio of 1:100 (*w*/*w*) and the samples were incubated at 37 °C for additional 4 h. The enzymatic reaction was stopped by acidification with 0.5% TFA (Sigma-Aldrich Inc., St.Louis, MO, USA), incubation at 37 °C for 45 min and centrifugation at 13,000× *g* for 10 min.

Before MudPIT analysis, samples were desalted by PepClean C-18 spin colums (Pierce Biothecnology Inc., Rockford, IL, USA), concentrated in a SpeedVac (Savant Instruments, Farmingdale, NY, USA) at 60 °C and finally resuspended in 0.1% formic acid (Sigma-Aldrich Inc., St.Louis, MO, USA).

### 2.3. MudPIT Analysis

Trypsin-digested samples were analyzed by means of Multidimensional Protein Identification Technology (MudPIT), using the ProteomeX configuration (Thermo Fisher, San Jose, CA, USA). Briefly, 10 μL of peptide mixture per sample were loaded into a strong cation exchange (SCX) column (Biobasic-SCX, 0.32 i.d.x100 mm, 5 μm, Thermo Scientific, Bellefonte, PA, USA) and eluted stepwise by increasing the ammonium chloride molarity (0, 20, 40, 80, 120, 200, 400, 700 mM NH_4_Cl). Eluate from each salt step was then captured on-line to be loaded into a reverse-phase (RP) C18 column (Biobasic-18, 0.180 i.d.x100 mm, 5 μm, Thermo Scientific, Bellefonte, PA, USA) and separated with an acetonitrile gradient (eluent A, 0.1% HCOOH in H_2_O; eluent B, 0.1% HCOOH in CH_3_CN). The eluted peptides were finally analyzed by an ion trap mass spectrometer (LTQ, Thermo Fischer, San Jose, CA, USA), equipped with a nano electrospray ionization source, collecting MS and MS/MS spectra.

### 2.4. Processing of Experimental Tandem Mass Spectra

The experimental tandem mass spectra (MS/MS) were matched against in silico spectra from the non-redundant *Pseudomonas aeruginosa* protein database (5564 entries), retrieved from UNIPROT (http://www.uniprot.org/) in June 2018. Data processing was performed by Discoverer2.1 software equipped with SEQUEST HT algorithm [20]. No enzyme specificity was assumed to identify semi- and not-tryptic peptides, too. The following criteria were used for peptide identification (Figure S1): briefly, parent and fragment mass tolerance of 0.6 Da and 500 ppm, respectively, and missed cleavage sites per peptide was set to 2. Matches between spectra were only retained if they had a minimum Xcorr of 2.0 for +1, 2.5 for +2, and 3.5 for +3 charge state, respectively, protein rank was fixed to 1, while peptide confidence was set to “high.” In addition, false discovery rate (FDR) was set to ≤1%.

### 2.5. CHARACTERIZATION of Trans-Membrane and Membrane-Exposed peptides

Experimental data here produced (GMF, *n* = 7; IM, *n* = 6; OM, *n* = 5) were combined with other data previously obtained by shaving intact *Pseudomonas aeruginosa* cells (SH, *n* = 6) and by using carboxymethyl-dextran coated magnetic nanoparticles (NP, *n* = 6) [9]. The identified proteins were ranked based on their abundance estimated by ntaSpC, the total average spectral count normalised on protein length (total aSpC/Lenght(aa))*1000). The data matrix reporting all analyzed samples and all identified peptides was processed by means of Linear Discriminant Analysis (LDA), using a common covariance matrix for all groups and the Mahalanobis distance from each point to each group’s multivariate mean [18]. To discriminate the peptides specifically enriched by the adopted extraction methods a F-ratio ≥ 3 and a *p*-value ≤ 0.05 were applied. Hierarchical clustering and centroid distance method were used to verify the discrimination level obtained by the peptides selected using LDA. To characterize trans-membrane and membrane-exposed peptides, and to increase their confidence, the peptides lists were grouped according to the extraction method applied (IM, OM, GMF, NP, SH), thus based on the protein portions they enriched. As a rule, peptides enriched by GMF, NP and SH were considered membrane-exposed, while those specifically enriched by IM or OM were classified as trans-membrane. Specifically, the identification by GMF or NP was a sufficient condition to consider a peptide membrane exposed, while the identification by SH was a necessary condition to define a peptide exposed at the external cell surface. Finally, an enrichment by IM or OM was indicative of an inner- or outer-membrane peptide, respectively. All processing were performed by in-house R scripts and JMP15 software.

### 2.6. Functional Classification, Amino Acid Distribution, Gravy Index, Immunogenicity and Antigenicity

Functional classification (enriched subcellular localization, biological processed and molecular functions) of the identified *Pseudomonas aeruginosa* proteins was performed by Panther database [21]; FDR ≤ 0.05 and Fisher’s Exact Test ≤ 0.05 were applied. Amino acid composition of trans-membrane and membrane-exposed peptides was compared by Chi-Square test (*p* < 0.05). Gravy index was calculated according to Kyte and Doolittle by using an in-house python script based on protparam library inserted in Biopython [22], while a model of prediction was used to estimate the peptide immunogenicity [23]. The average Gravy index and immunogenicity were compared by ANOVA and Tukey’s test. In addition, a non parametric test, such as Wolcoxon, was applied, too. To perform all statistical analyses in-house R scripts were used; in particular, tidyr and dplyr library were used for data transformation, ggplot2 library was used to make charts, while mvnor mtest library for evaluating the normal distribution of data by Shapiro–Wilks test. Finally, OM annotated proteins were evaluated about their antigenicity using VaxiJen server [24]. The proteins that contained the score value > 0.8 were considered for afterward analysis as an antigenic OMPs.

### 2.7. T Cell MHC (Class I and Class II) Epitope Binding and Processing Prediction

Antigenic OMPs selected using VaxiJen server (score > 0.8) were further evaluated about different T-cell epitope analysis for potent epitopes identification. T cell MHC Class I epitope binding and processing prediction was performed by NetMHCpan4.1 [25] and IEDB MHC-I [26] prediction server, respectively. For both analyses we selected a HLA supertype representative reference list that contained 12 different alleles. Other parameters of prediction tools were set to default. The top predicted epitopes from all the proteins were selected. About NetMHCpan4.1, only epitopes classified as Strong Binding (SB) were considered, while for IEDB MHC-I only ones with a total score > 1. Similarly, T cell MHC Class II epitope binding and processing prediction was performed by MHC-II binding (IEDB server) [27] and MHCII-NP [28] prediction server, respectively; seven-allele HLA reference set and a epitope length of 15 were set for binding prediction, while predicted epitopes were selected by considering binding and cleavage percentile rank (<0.1). B-cell epitope analysis for potent epitopes identification was performed by BepiPred-2.0 tool of IEDB server by applying default parameters [29]. Finally, selected epitopes were evaluated for their toxicity behaviour by ToxinPred tool [30]; specifically, Support Vector Machine (SVM) based method and E-value cut-off of 10 were applied.

## 3. Results

**Figure 1:**
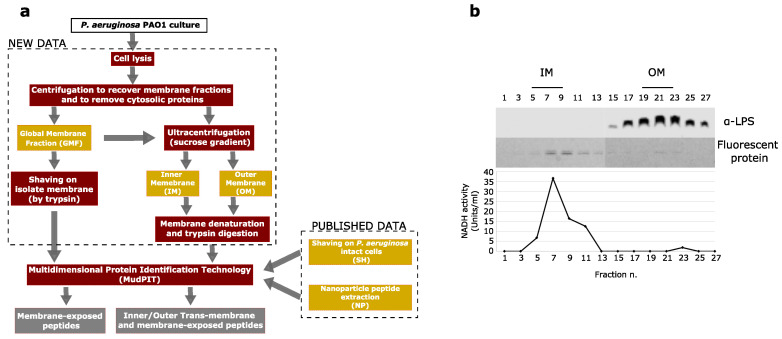
(**a**) Workflow for extracting and enriching exposed and embedded peptides from *P. aeruginosa* inner (IM) and outer (OM) membranes. GMF: global membrane fraction. SH: Shaving on intact *P. aeruginosa* cells. NP: peptide/protein extraction by Nanoparticles. (**b**) *P. aeruginosa* inner (IM) and outer (OM) membranes separated by sucrose gradient ultracentrifugation. IM and OM containing fractions were verified by the profile of NADH oxidase activity and distribution of LPS, respectively.

To characterize the landscape of *Pseudomonas aeruginosa* membrane-anchored proteins, *P. aeruginosa* global, inner and outer membranes were treated by different methods and selectively extracted (Figure 1). Global membrane fractions (GMFs) were "shaved" by trypsin for enriching membrane-exposed peptides, while IM and OM, separated by ultracentrifugation in a sucrose gradient, were first denatured and then enzymatically digested to extract both trans-membrane and membrane-exposed peptides. Peptides obtained following digestion were here identified by Multidimensional Protein Identification Technology (MudPIT). In addition, with the purpose to increase the proteome coverage, the experimental data produced here were integrated with others previously obtained by shaving intact *P. aeruginosa* cells (SH) and by using carboxymethyl-dextran coated magnetic nanoparticles (NP) [9].

### 3.1. Pseudomonas aeruginosa Membrane Proteome

**Figure 2:**
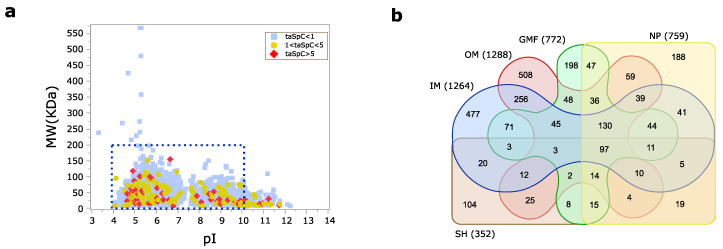
Multidimensional Protein Identification Technology (MudPIT) analysis of *P. aeruginosa* cell envelope. (**a**) Virtual 2D map of *P. aeruginosa* proteins identified by MudPIT approach (*n* = 2539 proteins). Light blue/square indicates proteins identified by a total average SpC ≤ 1, yellow/circle proteins by a total average SpC in the range 1–5, while red/rhombus proteins by a total average SpC ≥ 5. The blue box corresponds to the typical pI and MW ranges of 2D gel analysis. (**b**) Venn diagrams reporting the distribution of proteins identified following IM (Inner Membrane), OM (Outer Membrane), GFP (Global Membrane Fraction), SH (Shaving) and NP (Nanoparticles) extraction methods.

Following the combination of 29 MudPIT runs (incuding new and already published data), 10,611 unique peptides corresponding to 2539 distinct proteins were identified (Table S1). About 10% of proteins were characterized by an average spectral count (SpC) greater than 1 (Figure 2a). This seemingly low number was determined by the application of methods for enriching specific protein portions, thus reducing a wider sequence coverage and the possibility to identify a high number of peptides per protein. Starting from a comparable number of analyzed samples per condition, most proteins and peptides were found in IM and OM samples, while a lower number was identified by NP and GMF, and even less by SH (Figure 2b).

**Figure 3:**
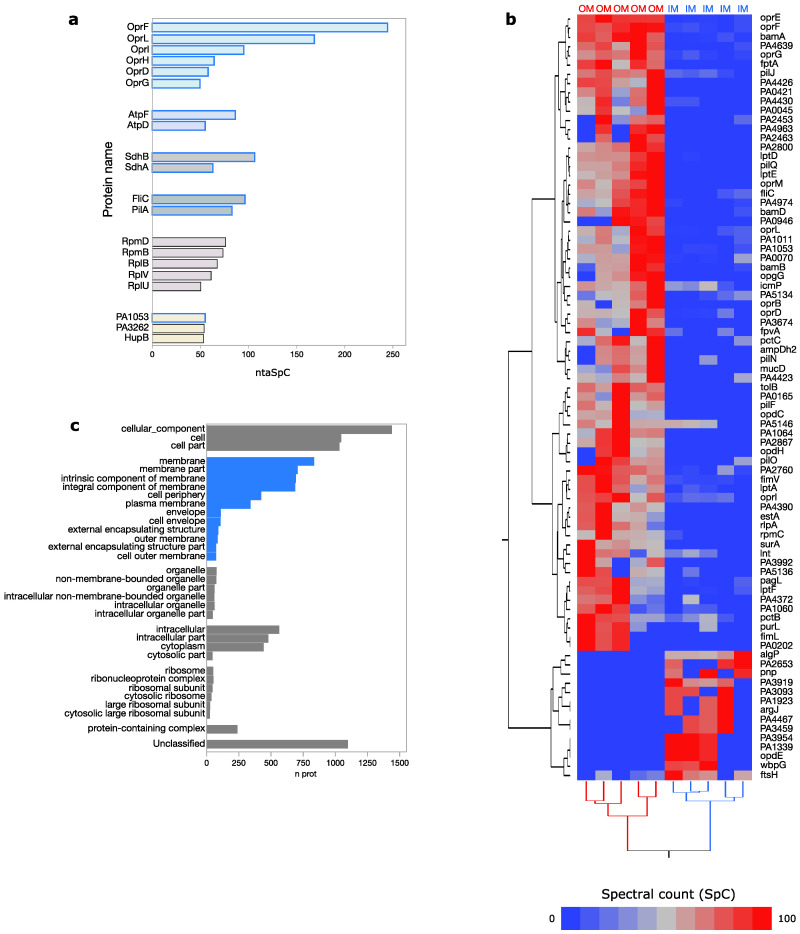
(**a**) Histogram showing the 20 most abundant proteins of *P. aeruginosa* envelope ranked based on total average spectral count normalized on protein length (ntaSpC). Blue border indicates proteins located in membrane. (**b**) Proteins differentially enriched between inner (IM) and outer membrane (OM). Proteins were extracted by Linear Discriminant Analysis (LDA), *p* < 0.05); hierarchical clustering performed by Ward’s methods and Euclidean distance (JMP15.1 software) by processing Spectral Count (SpC) of identified proteins. (**c**) Cellular components enriched from the characterized *P. aeruginosa* membrane proteome (*n* = 2539). The number of proteins per sub-cellular component is shown. Blue bars indicate cellular components related to membranes. Data were retrieved from Panther (www.pantherdb.org); FDR ≤ 0.05, Fisher’s Exact test ≤ 0.05.

Protein extraction methods we applied allowed enrichment of membrane-anchored proteins which were identified by a high number of peptides. Outer membrane porins (OprF, OprL, OprI, OprH, OprD, OprG), the ATP synthase b and beta (AtpF, AtpD), the Succinate dehydrogenase B and flavoprotein subunits (SdhB, SdhA) and proteins involved in bacteria motility (FliC, PilA) resulted among the 20 most abundant-ranked proteins (Figure 3a). As expected, some of these proteins, including outer membrane porins, became highly enriched in the outer membrane (OM) in agreement with their subcellular localization, predicted or experimentally determined, while a lower number of proteins, including transporters (PA1339, OpdE, PA4467, PA2653) and integral component of membrane (PA1923, FtsH), were specifically found in the inner membrane (IM) (Figure 3b).

The enrichment of membrane-anchored proteins with transporter activity globally emerged by the gene ontology distribution associated with all identified proteins (Tables S2–S4). Despite subcellular localization of almost 50% of proteins resulted unknown, proteins predicted to be localized in membranes were those most represented (Figure 3c). A lower number was found for cytoplasmic and intracellular annotated proteins, while ribosomal proteins, although much less represented, were found enriched, too. Interestingly, some ribosomal subunits (RpmD, RpmB, RplB, RplV, RplU) were among the most abundantly-ranked proteins (Figure 3a).

### 3.2. Trans-Membrane and Membrane-Exposed Peptides Classification

**Figure 4:**
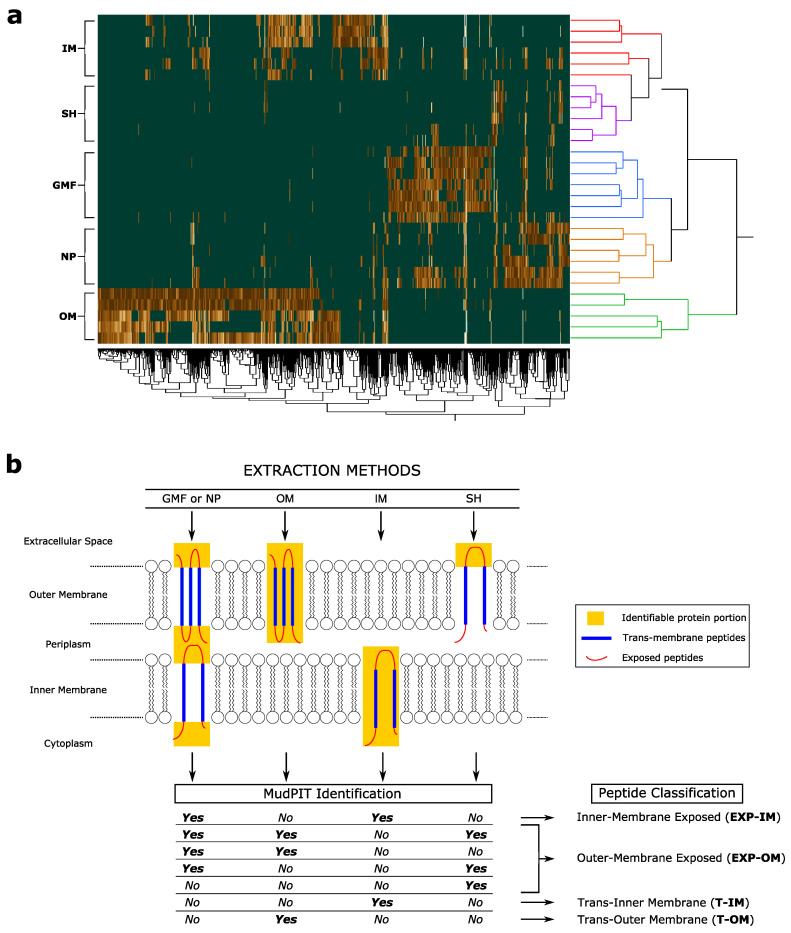
(**a**) Hierarchical clustering performed by computing the Xcorr values (cross-correlation between the experimental mass spectra and those theoretically predicted) of peptides (*n* = 722) specifically enriched following the extraction methods applied (LDA, *p* ≤ 0.01). Euclidean distance metric and Complete’s method were used. (**b**) Membrane protein portions (in yellow) enriched by IM (Inner Membrane), OM (Outer Membrane), GFP (Global Membrane Fraction), SH (Shaving) and NP (Nanoparticles). Combinations of MudPIT results used to classify peptides in Inner-Membrane Exposed (EXP-IM), Outer-Membrane Exposed (EXP-OM), Trans-Inner Membrane (T-IM) and Trans-Outer Membrane (T-OM).

In addition to characterizing *P. aeruginosa* membrane proteome as widely as possible, extraction methods applied aimed to discriminate specific protein portions. Based on that differential enrichment and according to the scheme shown in (Figure 4), peptides corresponding to 591 proteins were classified in Trans-Inner Membrane (T-IM, *n* = 248), Inner Membrane-Exposed (EXP-IM, *n* = 200), Trans-Outer Membrane (T-OM, *n* = 668) and Outer-Membrane Exposed (EXP-OM, *n* = 434), (Table S5); specifically, the identification of a given peptide following GFP/NP or SH extraction methods was the necessary condition to classify it as Exposed (EXP), while the exclusive identification by IM or OM methods was the necessary condition to classify peptides as Trans-Inner Membrane (T-IM) and Trans-Outer Membrane (T-OM), respectively.

**Figure 5:**
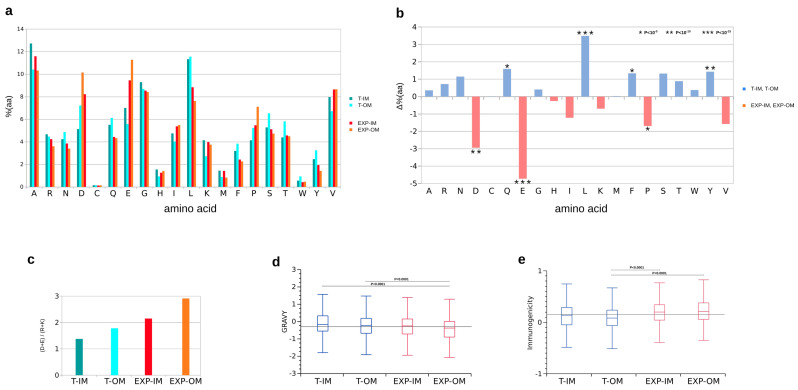
(**a**) Amino acid composition of T-IM, T-OM, EXP-IM, EXP-OM classified peptides. (**b**) Differences in amino acid composition (Δ%) between trans membrane (T-IM + T-OM) and exposed (EXP-IM + EXP-OM) classified peptides. Positive values (blue bars) indicate amino acids over-represented in trans membrane peptides, while negative values (red bars) indicated amino acids over-represented in exposed peptides. Data were compared by Chi-Square test ($p\leq 10^{-5}$). (**c**) Ratio between total number of negatively (D + E) and positively charged residues (R + K). (**d**) Comparison of Gravy index and (**e**) Immunogenicity calculated for T-IM, T-OM, EXP-IM, EXP-OM classified peptides (ANOVA and Tukey’s test; $p\leq 10^{-3}$).

The amino acid composition of selected peptide categories evidenced, in trans-membrane peptides, a higher representation of leucine (L), phenylalanine (F), tyrosine (Y) and Glutamine (Q). On the contrary, glutamic acid (E), aspartic acid (D) and proline (P) resulted over-represented in exposed peptides (Figure 5a,b). Exposed peptides showed also a ratio of negatively (D + E) and positively charged residues almost double of trans-membrane ones (Figure 5c). Statistically significant differences were also observed in Gravy index and Immunogenicity. As expected, Trans-Inner Membrane (T-IM) peptides showed the higher average Gravy value, while Outer-Membrane Exposed peptides (EXP-OM) the lowest. As opposite, Outer-Membrane Exposed peptides (EXP-OM) showed the higher value of Immunogenicity (Figure 5d,e).

### 3.3. Inner and Outer Membrane Classified Peptides and P. aeruginosa Protein Models

The accuracy of our peptide classification was tested using as a reference *Pseudomonas aeruginosa* proteins whose subcellular localization was predicted or experimentally determined in membrane (Table S6). In agreement with their subcellular localization, the outer membrane protein family (OprB, OprC, OprD, OprE, OprF, OprG, OprH, OprI, OprL, OprM) [31], the outer membrane protein assembly factors (BamA, BamB, BamD, BamE) [32], the Lpt complex (LptA, LptD, LptE) for LPS transport to the cell surface [19,33], as well as many other outer membrane annotated proteins, were predominantly characterized by outer-membrane classified peptides. On the contrary, the ATP synthase subunits [34,35], located in inner-membrane, by inner-membrane classified ones (Table 1).

### 3.4. Antigenicity, Immunogenicity and Toxicity of Selected Outer Membrane Proteins

**Figure 6:**
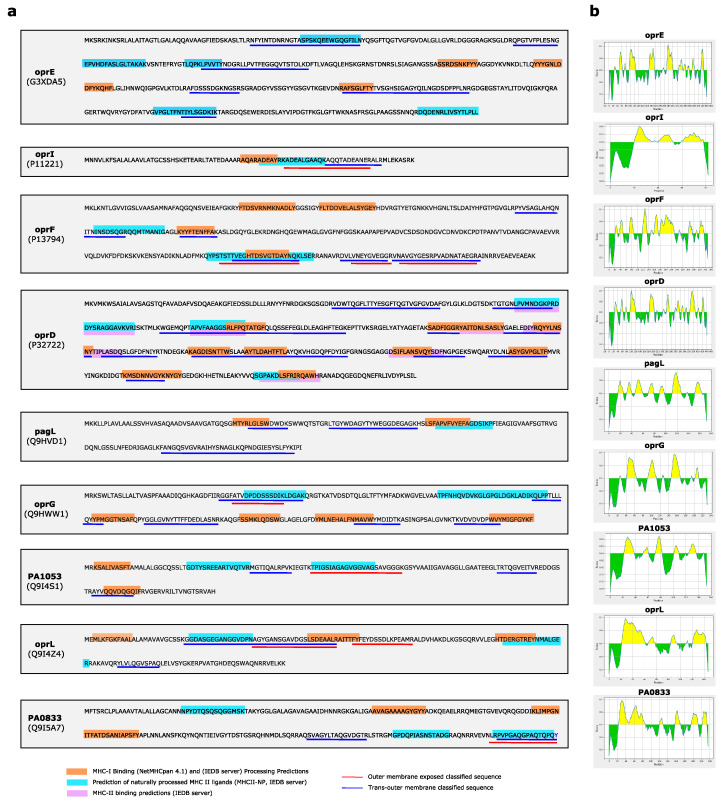
(**a**) Experimentally identified peptides, classified in EXP-OM or T-OM (red and blue lines, respectively), of oOM annotated proteins selected by a Vaxygen score > 0.8. Epitopes putatively bound and processed by T cell MHC Class I and Class II are shown; in orange are highlighted epitopes putatively bound and processed by MHC Class I, while in violet and blue those putatively bound and processed by MHC Class II, respectively. (**b**) Prediction of epitopes, in yellow, putatively bound and processed by B cells.

Since major potentially antigenic proteins of *P. aeruginosa* are outer-membrane exposed, all proteins outer-membrane annotated and characterized by OM classified peptides were processed by Vaxygen server to predict their antigenicity (Table S6). Following the application of stringent criteria of filtering (score < 0.8), 9 proteins, such as OprE, OprI, OprF, OprD, PagL, OprG, PA1053, PAL and PA0833, were selected and further investigated to predict which of their epitopes could be bound and processed by T cell MHC Class I, Class II and B cell (Figure 6). All selected epitopes resulted also non-toxic (Table S7). Based on parameters set and filters applied, most of them were predicted targets of MHC Class I or Class II, with few overlapping. oprD was the only protein whose epitopes were predicted to bind MHC Class II. Of note, epitopes matching with EXP-OM classified peptides were found for oprI and PA1053. In other cases, i.e., OprF, we found epitopes matching to sequences, partially overlapped, classified T-OM and EXP-OM. Following the scheme in Figure 4, these overlapped sequences should be considered exposed. However, concerning OprF these seemingly ambiguous results could be due to the two different conformers of OprF [36,37], further confirming the accuracy of our classification method. The predominant OprF conformer is constituted by a N-terminal β-barrel domain (aa 1-160) inserted into membrane and by a C-terminal periplasmic domain rich in alpha-helices (aa 161-312), while in a second one the C-terminal periplasmic domain is membrane-embedded, too (Figure S1).

## 4. Discussion

The present study aims to shed light on the landscape of *P. aeruginosa* membrane-anchored proteins. In fact, following the identification of 2539 distinct proteins, it represents one of the widest characterizations of the *P. aeruginosa* membrane proteome. Most of the proteins identified and provided with functional annotation were located in the membrane and involved in transport activity, highlighting the selectivity of the extraction methods applied. This aspect further emerged by the presence of several outer membrane porins among the most abundant identified proteins. Interestingly, also some ribosomal subunits were found abundant fitting with observations reported in previous studies [14,38,39]; however, their role in a membrane district has not yet been fully explored and further studies are needed to validate these observations and to elucidate the reasons for that.

Besides, to increase the membrane proteome coverage of *P. aeruginosa*, discerning from IM and OM, the combination of selective peptide and protein extraction methods had the purpose to get a differential enrichment of protein portions to discriminate in exposed and trans-membrane peptides. The proposed classification was in good agreement with the protein topology available and highlighted, as expected, a different tendency of the Gravy index and predicted immunogenicity. As conceivable, the variation of these indices maybe related to peptide chemical-physical properties, thus to their amino acid composition. In this context, trans-membrane classified peptides were characterized by the high percentage of leucine (L) that, thanks to its alkyl side-chain, confers to peptides non-polar characteristics fitting with their positioning in membrane bilayer. Similarly, tyrosine (Y) and phenylalanine (F) may favor anchoring of proteins into the membrane through the interaction of their aromatic rings with the lipid head groups [40,41], while glutamine has been put in relation with self-association of transmembrane β-peptides within lipid bilayers [42]. Differently, as for exposed classified peptides, the higher presence of aspartic (D) and glutamic (E) acids fits with their positioning in aqueous environment: in fact, being negatively charged and polar amino acids, they prefer generally to be on the protein surface [43]. Furthermore, although less significant, prevalence of proline residues in exposed peptides could be related to their ability in generating loop. The role of this amino acid has been correlated to immunomodulation as well as to protein–protein interactions including host-pathogen recognition [44,45]. It is well known that outer membrane proteins play a relevant role in these mechanisms [46]. Thus, a recent work highlighted their potential in vaccine development [47]. In agreement with these findings, the potential antigenicity of nine outer membrane proteins, including several outer membrane porins, emerged fron our study. Some combination of these proteins, including OprF and OprI [48], or OprF and OprL [49], are currently investigated as vaccine candidates. Similarly, PA0833 was demonstrated is an OmpA C-like protein that induces a protective immune response in mice, indicating it as a promising antigen for vaccine development [50].

Starting from the promising results we obtained, in the next future the proposed strategy will have to be applied to *P. aeruginosa* at different conditions, mimicking infection models and affecting the expression of membrane exposed proteins. In fact, it has recently demonstrated that the expression of some proteins, including outer-membrane ones, may be affected by the environmental conditions. For instance, TonB-dependent transporters (TBDTs) present in the outer membranes of *P. aeruginosa* are induced under iron restricted conditions or when cells have been harvested from an infection model [51]. Although bacteria we analyzed in our work were grown in Mueller Hinton (MH) medium, far from the conditions adopted by Perraud et al., many TBDTs were identified also in our samples. These proteins were here characterized by low identification frequency and low normalized average spectral counts (ntaSpC), in agreement with an expression level generally low [52]. This low level could therefore reasonably be associated with a bacterial environment, highlighting the need to investigate *P. aeruginosa* membrane-associated proteins at different conditions. Finally, other aspects of our study may certainly be improved. A weak point may be related to the low capacity of the proposed approach in classifying periplasmic peptides. However, we already highlighted that the goal of our manuscript was to provide a landscape of *P. aeruginosa* membrane-anchored proteins discriminating between inner and outer membrane. On the other hand, the validation of our approach by comparing the obtained results with the subcellular localization of the classified peptides/proteins remains an open question. In fact, many annotations are computationally predicted bringing not always to correct considerations.

## 5. Conclusions

The present study provides a wide listing of *P. aeruginosa* membrane-associated proteins and represents a proof of principle to develop genome-scale high-throughput methods well-suited to define membrane protein topology. The feasibility of this approach may facilitate its use for investigating other bacterial species, especially those highly pathogenic whose envelope exposed protein domains are still unclear. In addition to describe the landscape of *P. aeruginosa* membrane-associated proteins, we provided also an ensemble of proteins that could induce immunogenicity and feasibility of a vaccine construct; many of those tested in preclinical trials did not reach the clinical phase, and none have been approved for marked authorization [53]. Thus, the data here presented suggest that stepwise prioritization of proteome, obtained by combining experimental proteomic data and bioinformatic approaches, may be useful for reducing the number of proteins that could be tested in vaccine development.

## Figures and Tables

**Table 1:** Classification of peptides from *P. aeruginosa* proteins localized in membranes. The number of inner- and outer-membrane classified peptides per protein is highlighted in yellow and blue, respectively. T-IM: Trans-Inner Membrane, T-OM: Trans-Outer Membrane, EXP-IM: Inner-Membrane Exposed, EXP-OM: Outer-Membrane Exposed; full data reported in Table S6.

UNIPROT ID	Protein Name	Gene Name	EXP-IM	T-IM	EXP-OM	T-OM	Sub. Localization
Q51485	Porin B	oprB				4	plasma membrane [GO:0005886]
G3XD89	Putative copper transport outer membrane porin OprC	oprC			-	5	plasma membrane [GO:0005886]
P32722	Porin D	oprD				18	plasma membrane [GO:0005886]
G3XDA5	Anaerobically-induced outer membrane porin OprE	oprE				14	plasma membrane [GO:0005886]
P13794	Outer membrane porin F	oprF		1	5	11	plasma membrane [GO:0005886]
Q9HWW1	Outer membrane protein OprG	oprG			1	10	plasma membrane [GO:0005886]
G3XD11	PhoP/Q and low Mg2+ inducible outer membrane protein H1	oprH	2	3	1	1	plasma membrane [GO:0005886]
P11221	Major outer membrane lipoprotein	oprI			1	2	plasma membrane [GO:0005886]
Q9I4Z4	Peptidoglycan-associated lipoprotein	oprL	1	2	3	9	plasma membrane [GO:0005886]
Q51487	Outer membrane protein OprM	oprM		-	-	15	plasma membrane [GO:0005886]
Q9HXY4	Outer membrane protein assembly factor BamA	bamA		2	1	19	cell outer membrane [GO:0009279]
Q9HXJ7	Outer membrane protein assembly factor BamB	bamB			2	8	cell outer membrane [GO:0009279]
P33641	Outer membrane protein assembly factor BamD	bamD				6	cell outer membrane [GO:0009279]
O68562	Outer membrane protein assembly factor BamE	bamE			1	3	cell outer membrane [GO:0009279]
Q9HVV7	Lipopolysaccharide export system protein LptA	lptA				1	cell outer membrane [GO:0009279]
Q9I5U2	LPS-assembly protein LptD	lptD				23	cell outer membrane [GO:0009279]
Q9HX32	LPS-assembly lipoprotein LptE	lptE		1		4	cell outer membrane [GO:0009279]
Q9HT18	ATP synthase subunit alpha	atpA	10	1	2	-	plasma membrane [GO:0005886]
Q9HT14	ATP synthase subunit a	atpB	1	1	-	-	plasma membrane [GO:0005886]
Q9HT21	ATP synthase epsilon	atpC	1	1	-	-	plasma membrane [GO:0005886]
Q9HT20	ATP synthase subunit beta	atpD	11	6	-	1	plasma membrane [GO:0005886]
Q9HT16	ATP synthase subunit b	atpF	-	2	-	-	plasma membrane [GO:0005886]
Q9HT19	ATP synthase gamma	atpG	3	-	-	-	plasma membrane [GO:0005886]

## Supplementary Materials

The following are available at https://www.mdpi.com/2073-4409/9/11/2421/s1, Figure S1: Predominant conformer of oprF, Table S1: Proteins identified by analyzing *P. aerugnosa* cells treated by IM (*n* = 6), OM (*n*= 5), GFP (*n* = 6), NP (*n* = 6), SH (*n* = 6) protein extraction methods, Table S2: Cellular components associated with the *P. aeruginosa* membrane proteome characterized by IM, OM, GFP, SH and NP extraction methods, Table S3: Molecular functions associated with the *P. aeruginosa* membrane proteome characterized by IM, OM, GFP, SH and NP extraction methods, Table S4: Biological processes associated with the *P. aeruginosa* membrane proteome characterized by IM, OM, GFP, SH and NP extraction methods, Table S5: Classification of experimentally identified peptides in trans inner membrane (T-IM), inner membrane exposed (EXP-IM), trans outer membrane (T-OM) and outer membrane exposed (EXP-OM), Table S6: Classification of experimentally identified peptides for proteins localized in *P. aeruginosa* plasma membrane, Table S7: Prediction of epitopes putatively bound and processed by T cell MHC Class I (NetMHCpan 4.1 and MHC-I Processing Predictions) and Class II (MHC-II binding predictions and MHCII-NP).

## Abbreviations

The following abbreviations are used in this manuscript:

IM	Inner Membrane
OM	Outer Memebrane
LC	Liquid Chromatography
MS	Mass Spectrometry
GFM	Global Membrane Fraction
MudPIT	Liquid Chromatography
SH	Shaving
NP	Carboxymethyl-dextran coated magnetic nanoparticles
SpC	Spectral Count
ntaSpC	total average Spectral Count normalized on protein length
LDA	Linear Discriminant Analysis
FDR	False Discovery Rate
T-IM	Trans Inner Membrane peptide
EXP-IM	Inner Membrane Exposed peptide
T-OM	Trans Outer Membrane peptide
EXP-OM	Outer Membrane Exposed peptide
MH	Mueller Hinton
OD	Optical Density
SCX	Strong Cation Exchange
RP	Reverse Phase
OMP	Outer Membrane Proteins
SVM	Support Vector Machine
TBDTs	TonB-dependent transporters
