# Supplementary material for: The Landscape of Pseudomonas aeruginosa Membrane-Associated Proteins
Source: Cells. 2020 Nov 5;9(11):2421. doi: 10.3390/cells9112421 (PMC7694347; doi:10.3390/cells9112421)
Supplement: Supplementary file 1 [file cells-09-02421-s001.zip › cells-976337-supplementary/Figure S1.pdf]

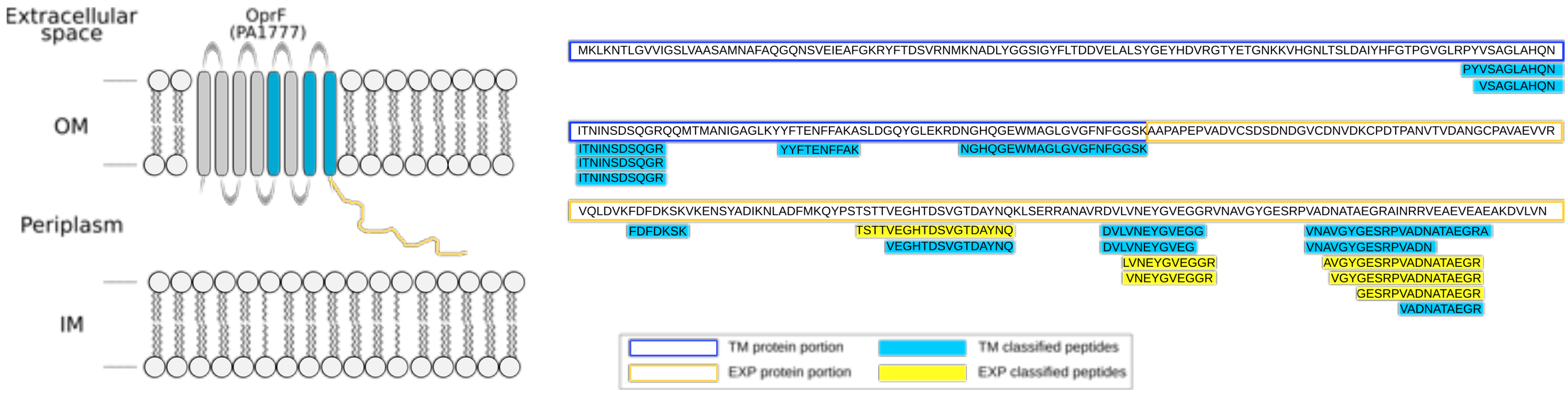

Figure S1. Predominant conformer of oprF. It is constituted by a N-terminal beta-barrel domain (aa 1-160), in blue, inserted into membrane and by a C-terminal periplasmic domain rich in alpha-helices (aa 161-312), in yellow. However, in a second conformer the C-terminal periplasmic domain is membrane embedded, too (Sugawara et al. mBio. 2010 Oct 19;1(4):e00228-10; Sugawara et al. FEBS J. 2012 Mar;279(6):910-8).
